# *Plasmodium* Topoisomerase VIB and Spo11 Constitute Functional Type IIB Topoisomerase in Malaria Parasite: Its Possible Role in Mitochondrial DNA Segregation

**DOI:** 10.1128/spectrum.04980-22

**Published:** 2023-05-22

**Authors:** Priyanka Singh, Wahida Tabassum, Nupur Fangaria, Sandeep Dey, Siladitya Padhi, Mrinal K. Bhattacharyya, Kota Arun Kumar, Arijit Roy, Sunanda Bhattacharyya

**Affiliations:** a Department of Biotechnology and Bioinformatics, School of Life Sciences, University of Hyderabad, Hyderabad, India; b Department of Biochemistry, School of Life Sciences, University of Hyderabad, Hyderabad, India; c Department of Animal Biology, School of Life Sciences, University of Hyderabad, Hyderabad, India; d TCS Research-Hyderabad (Life Sciences Division), Tata Consultancy Services Limited, Hyderabad, India; Hebrew University of Jerusalem

**Keywords:** *Plasmodium* mitochondria, *Plasmodium* topoisomerase VI, apicomplexan topoisomerase

## Abstract

The human malaria parasite undergoes a noncanonical cell division, namely, endoreduplication, where several rounds of nuclear, mitochondrial, and apicoplast replication occur without cytoplasmic division. Despite its importance in *Plasmodium* biology, the topoisomerases essential for decatenation of replicated chromosome during endoreduplication remain elusive. We hypothesize that the topoisomerase VI complex, containing Plasmodium falciparum topiosomerase VIB (PfTopoVIB) and catalytic P. falciparum Spo11 (PfSpo11), might be involved in the segregation of the *Plasmodium* mitochondrial genome. Here, we demonstrate that the putative PfSpo11 is the functional ortholog of yeast Spo11 that can complement the sporulation defects of the yeast Δ*spo11* strain, and the catalytic mutant Pfspo11Y65F cannot complement such defects. PfTopoVIB and PfSpo11 display a distinct expression pattern compared to the other type II topoisomerases of *Plasmodium* and are induced specifically at the late schizont stage of the parasite, when the mitochondrial genome segregation occurs. Furthermore, PfTopoVIB and PfSpo11 are physically associated with each other at the late schizont stage, and both subunits are localized in the mitochondria. Using PfTopoVIB- and PfSpo11-specific antibodies, we immunoprecipitated the chromatin of tightly synchronous early, mid-, and late schizont stage-specific parasites and found that both the subunits are associated with the mitochondrial genome during the late schizont stage of the parasite. Furthermore, PfTopoVIB inhibitor radicicol and atovaquone show synergistic interaction. Accordingly, atovaquone-mediated disruption of mitochondrial membrane potential reduces the import and recruitment of both subunits of PfTopoVI to mitochondrial DNA (mtDNA) in a dose-dependent manner. The structural differences between PfTopoVIB and human TopoVIB-like protein could be exploited for development of a novel antimalarial agent.

**IMPORTANCE** This study demonstrates a likely role of topoisomerase VI in the mitochondrial genome segregation of Plasmodium falciparum during endoreduplication. We show that PfTopoVIB and PfSpo11 remain associated and form the functional holoenzyme within the parasite. The spatiotemporal expression of both subunits of PfTopoVI correlates well with their recruitment to the mitochondrial DNA at the late schizont stage of the parasite. Additionally, the synergistic interaction between PfTopoVI inhibitor and the disruptor of mitochondrial membrane potential, atovaquone, supports that topoisomerase VI is the mitochondrial topoisomerase of the malaria parasite. We propose that topoisomerase VI may act as a novel target against malaria.

## INTRODUCTION

Malaria is a serious concern to public health. According to the latest WHO report (1), there were an estimated 241 million malaria cases in 2020 and 95% of these cases were reported in Africa. Alarmingly, 80% of malaria-related deaths in Africa occur in children under the age of 5 years. This necessitates the importance of basic research with malaria parasites to identify novel proteins that can act as an antimalarial target. Topoisomerases pose an attractive antimalarial target due to the absence of some of the unique topoisomerases from the human genome (2).

The malaria parasite undergoes a noncanonical cell division known as endoreduplication: twice in the human host and once in the mosquito midgut. During this cell cycle, multiple rounds of genome replication occur without cytokinesis. An earlier study has shown that in Arabidopsis thaliana, topoisomerase VI (TopoVI) is essential for decatenation of the replicated chromosome during endoreduplication (3). It was reported that TopoVI deletion mutants can complete only first two endocycles and stall at 8C, compared to the wild-type cells that complete four rounds of endoreduplication and display 32C (3). *Plasmodium* possesses topoisomerase VI, but whether it is involved in the segregation of the parasite genome during endoreduplication remains elusive. Live-cell imaging of Plasmodium falciparum revealed that while the nuclear and apicoplast division occur in the early and mid-schizont stages, respectively, the mitochondrial division happens shortly before the cell division (4). It can be speculated that the mitochondrial genome segregation is initiated in the late schizont stage of the parasite.

Earlier we have shown that *Plasmodium* topoisomerase VI can genetically complement topoisomerase II function in Saccharomyces cerevisiae. Ectopic expression of P. falciparum TopoVI (PfTopoVI) was found to rescue a Δ*topoII* lethal mutant strain (5). Using the cell extract of Δ*topoII* yeast harboring PfTopoVI, we have shown that it can decatenate the kinetoplast DNA. Although PfTopoVI has a type II topoisomerase activity, direct demonstration of its precise function in the malaria parasite has not been done so far.

Topoisomerase VI was first characterized in Sulfolobus shibatae (6) and then in plants (3). In plants and algae, TopoVI has two subunits, TopoVIA and TopoVIB, which together form the functional enzyme. While TopoVIA harbors the DNA binding and DNA cleavage domain, TopoVIB harbors the GHKL domain responsible for ATP binding and ATP hydrolysis. It was demonstrated that ATP binding to the TopoVIB is essential for stabilization of TopoVI enzyme, which is required for DNA cleavage (7). The eukaryotic orthologue of TopoV1A is known as Spo11 (8). *Plasmodium* harbors both subunits of PfTopoVI, namely, PfSpo11 (new ID, PF3D7_1217100) and PfTopoVIB. In our earlier study, PfSpo11 was referred to as PfTopoVIA (old ID, PF3D7_1217100.1) (5). Here, we have referred to this protein as PfSpo11. Using a yeast two-hybrid assay, it was shown earlier that PfTopoVIA and PfTopoVIB associate with each other. Furthermore, using yeast cell extract harboring PfTopoVI, it was shown that the decatenation activity of the enzyme is inhibited by radicicol; this was observed at sublethal doses, Radicicol reduces the mitochondrial genome content of the parasite (9), but the mechanism of such an effect was not investigated. Furthermore, whether PfSpo11 and PfTopoVIB form the functional holoenzyme within the parasite mitochondria remains elusive. In addition, whether PfSpo11 is the catalytic subunit of TopoVI enzyme was not investigated.

In this study, we have established for the first time that PfSpo11 is the functional ortholog of yeast Spo11. We provide evidence for the existence of PfTopoVI holoenzyme within the mitochondria of malaria parasite and its probable function in mitochondrial genome segregation. Our study was further supported by the result that the PfTopoVIB inhibitor radicicol synergizes with atovaquone, an antimalarial drug that collapses mitochondrial membrane potential, within the malaria parasite.

In humans, TopoVIB orthologs are not found, albeit human TopoVIB-like protein (TopoVIBL) has been identified, which shows 10% identity with *Plasmodium* TopoVIB. The ATPase domain of PfTopoVIB is distinct from the canonical ATP binding fold (Walker ATPases) and known as the Bergerat fold, which is characterized by four signature boxes: N, G1, G2, and G3. We found that there is no similarity between the predicted structures of the Bergerat folds of human TopoVIBL and *Plasmodium* VIB protein.

## RESULTS

### PfSpo11 complements the sporulation defect of a diploid Δ*spo11* strain.

**FIG 1:**
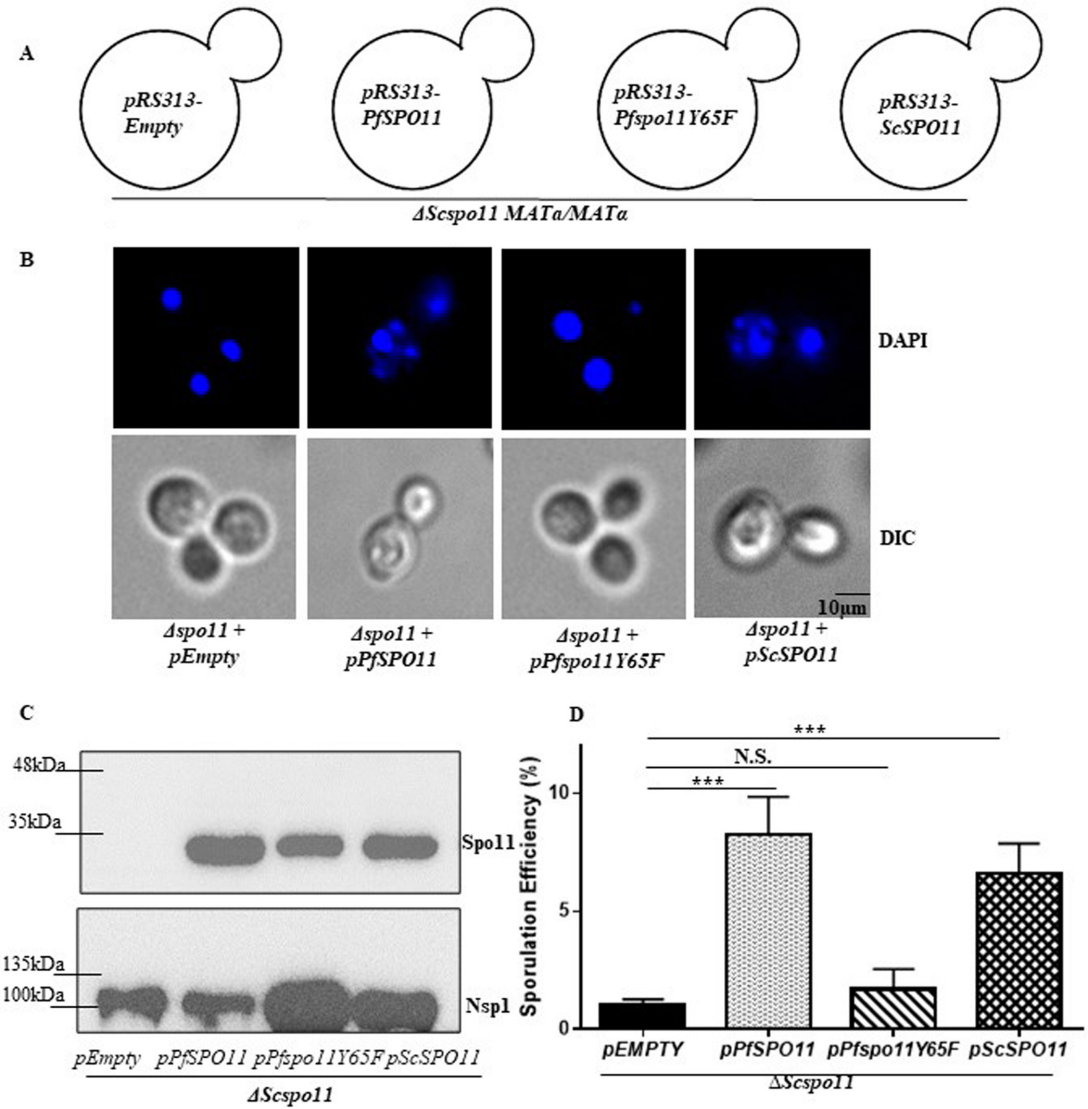
PfSpo11 complements the sporulation defect of the diploid Δ*spo11* strain. (A) Schematic representation of four diploid yeast strains generated for the sporulation assay: Δ*Scspo11 MAT***a***/MATα-Empty pRS313*, Δ*Scspo11 MAT***a***/MATα-pRS313-PfSPO11*, Δ*Scspo11 MAT***a**/*MATα-pRS313-Pfspo11Y65F*, and Δ*Scspo11 MAT***a**/*MATα-pRS313-ScSPO11*; (B) fluorescence imaging of the respective diploid strains that were subjected to sporulation. The cells were stained with DAPI to visualize the nuclei. The strain expressing *PfSPO11* can complement the sporulation defect of Δ*Scspo11* strains; however, the strain expressing *Pfspo11Y65F* cannot. DIC, differential inference contrast. (C) Western blot showing the expression of Spo11 in the respective strains. Nsp1 was used as a loading control. (D) For each strain, we counted the number of cells that can form mature asci (3 or 4 spores), and the percentage of sporulation was calculated. The experiment was repeated with three independent batches of cells for each strain (*n* = 1,000 cells). The mean values ± SD were plotted for each strain using GraphPad Prism 6. *P* values were calculated using two-tailed Student's *t* test (***, *P* < 0.001; N.S., not significant).

To evaluate the function of PfSpo11, we tried to express the recombinant PfSpo11 protein in various bacterial systems (5); however, we were not successful, and hence we used yeast as a surrogate system. Diploid budding yeast undergoes meiosis in response to nitrogen starvation, and the haploid nuclei generated during meiosis are packaged in spores. The meiosis is initiated by Spo11, which catalyzes the cleavage of double-stranded DNA, which is subsequently repaired by recombination between the parental chromosomes. In plants, Spo11 along with TopoVIB forms the functional complex; however, in yeast, TopoVIB being absent, yeast Spo11 (ySpo11) can catalyze the double-strand break (DSB) formation by interacting with several other proteins (10). It was reported earlier that diploid cells with Spo11 deleted display reduced efficiency of sporulation due to the defect in meiotic recombination (11). We examined whether putative PfSpo11 can complement the function of S. cerevisiae Spo11 (ScSpo11). PfSpo11 harbors the conserved DNA binding CAP (catabolite activating protein) domain and metal binding TOPRIM domain, which share 42.7% and 59.5% sequence similarity, respectively, with the corresponding domains of ScSpo11 (5). We cloned *PfSPO11* and *ScSPO11* in the centromeric yeast expression vectors. Using site-directed mutagenesis, we generated a point mutation (Y to F) to the catalytic tyrosine residue of *PfSPO11* at the 65th position with phenylalanine. For our assay, we used the diploid Δ*spo11* strain (BY4741), which is of S228C origin and was earlier reported to show 5 to 15% sporulation efficiency (12). We generated four isogenic strains, each harboring empty vector (negative control), *PfSPO11*, *Pfspo11Y65F*, and *ScSPO11* (positive control), as presented schematically in Fig. 1A. The sporulation was induced in these strains by growing them in presporulation liquid medium for 18 h at 30°C, and we subsequently allowed them to grow for another 48 h in sporulation medium at 18°C. The spore formation was visualized under a fluorescence microscope after staining with DAPI (4′,6-diamidino-2-phenylindole) (Fig. 1B). It was observed that *PfSPO11*-expressing strain can bypass the sporulation defect of the Δ*Scspo11* strain and produces a comparable number of spores to that of the strain expressing *ScSPO11*. *PfSPO11*- or *ScSPO11*-expressing strains formed 4 distinct nuclei, as represented in the figure; however, the catalytic mutant failed to sporulate. In order to rule out the possibility that the loss of sporulation in the PfSpo11Y65F strain is due to loss of expression of the mutant protein in yeast, we performed a Western blot analysis with the proteins extracted from each of the strains. Our study confirmed the expression of PfSpo11 and the mutant in the respective strains (Fig. 1C). We measured the sporulation efficiencies with three independent batches of cells in each strain and counted more than 1,000 cells in each case; the data are presented in Fig. 1D. We observed that PfSpo11 can complement the sporulation defect of the Δ*spo11* strain (1.1%) to the same extent as ScSpo11. Our analysis showed that the *PfSPO11*-harboring strain showed a sporulation efficiency (8.3%) similar to that obtained by the *ScSPO11*-harboring strain (6.6%); however, the catalytic mutant *Pfspo11Y65F* strain showed a severe defect in sporulation as there was a drastic reduction in its efficiency (1.7%), which was comparable to that of the Δ*spo11* strain. Thus, our study shows for the first time that PfSpo11 is the functional ortholog of ySpo11 and its activity is dependent on the catalytic tyrosine residue.

### PfTopoVIB and PfSpo11 display a unique expression compared to the other type II topoisomerases of P. falciparum.

**FIG 2:**
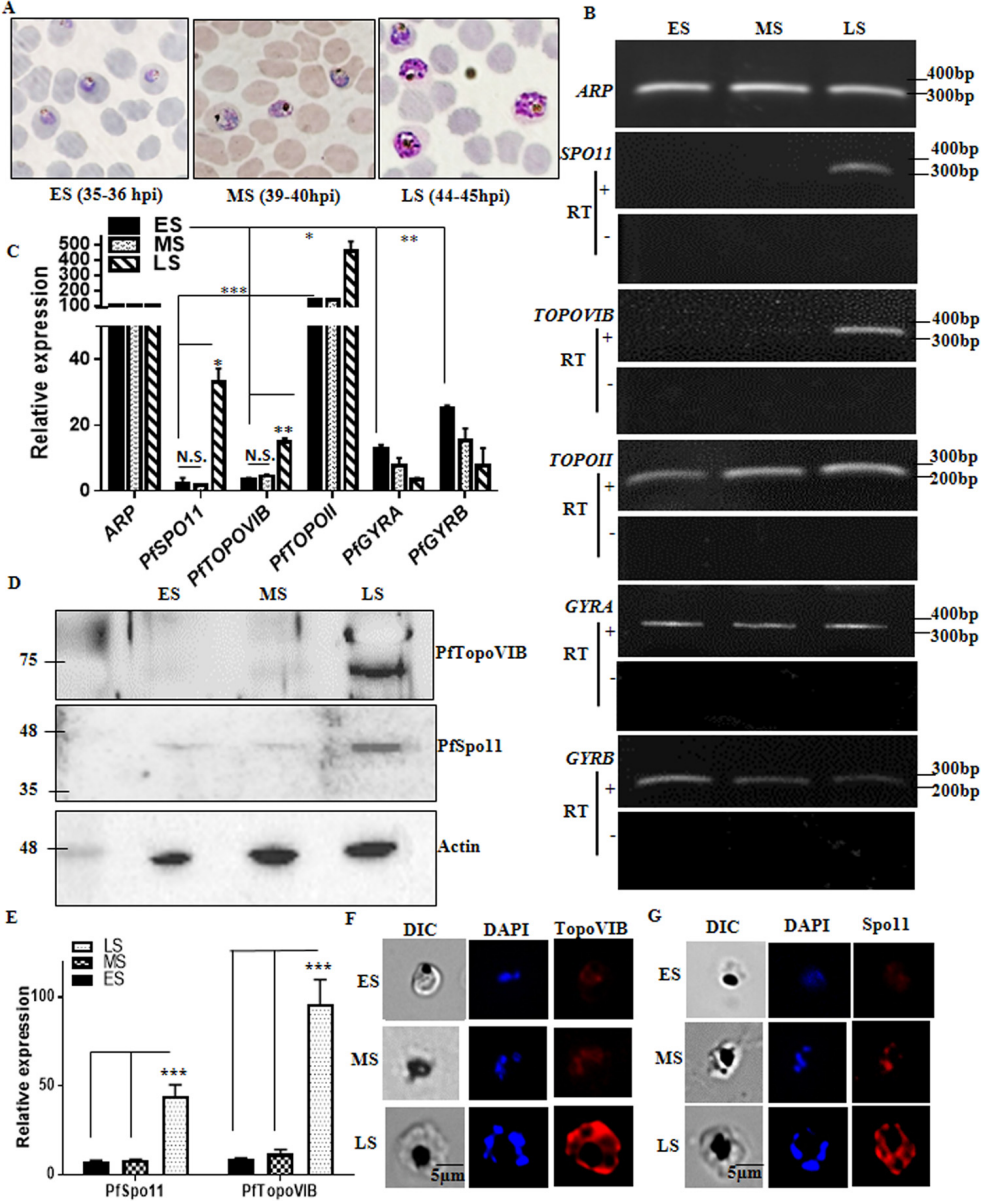
PfTopoVIB and PfSpo11 display unique expression compared to the other type II topoisomerases of P. falciparum. (A) Giemsa-stained representative images of the synchronized ES (early schizont [35 to 36 hpi]), MS (mid-schizont [39 to 40 hpi]), and LS (late schizont [44 to 45 hpi])-specific 3D7 parasites; (B) semi-qRT-PCR analysis was done from the cDNA prepared from the ES, MS, and LS stages of the parasites to visualize the expression of all type II topoisomerases (*PfTOPOII*, *PfGYRA*, *PfGYRB*, *PfTOPOVIB*, and *PfSPO11*). Agarose gel images for both positive and negative reverse transcriptase (+ RT and – RT, respectively) samples are presented. (C) Real-time RT-PCR analysis was done to quantify the relative expression of all of the type II topoisomerases in comparison to those constitutively expressing *ARP* at the ES, MS, and LS stages. The experiment was repeated with two independent batches of parasites. *P* values were calculated using two-tailed Student's *t* test (***, *P* < 0.001; **, *P* < 0.01; *, *P* < 0.05; N.S., not significant). (D) Western blot analysis shows that PfTopoVIB and PfSpo11 are expressed predominantly at the LS stages of the parasite; actin was used as a normalizing control. (E) Relative levels of protein expression of PfTopoVIB and PfSpo11 were calculated using ImageJ from three independent batches of synchronized ES, MS, and LS stage-specific parasite proteins, and the mean values ± SD were plotted. *P* values were calculated using the two-tailed Student's *t* test (***, *P* < 0.001). (F and G) Indirect immunofluorescence images showing the expression of PfTopoVIB and PfSpo11 predominantly at the LS stages. DAPI was used to stain the nucleus.

Parasite genome replication is initiated at the late trophozoite/early schizont stage of the parasite, and hence the type II topoisomerases that remove the topological strains for the progression of the replication fork need to be expressed during the initiation of DNA replication. Earlier we found that the two subunits of PfTopoVI were predominantly expressed in the schizont-specific stages compared to the ring and trophozoite stages, respectively (9). In order to have a better understanding of the function of PfTopoVI, we measured the transcripts of other type II topoisomerase subunits identified in the parasite (2) at three distinct developmental stages within the schizont. We used tightly synchronized parasites 35 to 36 h postinvasion (hpi) as early schizont (ES) stage-specific, 39 to 40 hpi as mid-schizont (MS) stage-specific, and 44 to 45 hpi as late schizont (LS) stage-specific parasites for all of our experiments (Fig. 2A). These stages were carefully chosen by examining the relative size of the nucleus under a microscope as shown in the representative pictures. The semiquantitative reverse transcription-PCR (semi-qRT-PCR) data showed that *PfTOPOVIB* and *PfSPO11* displayed a unique expression pattern, unlike other type II topoisomerase genes, and were not expressed in the ES and MS stages (Fig. 2B). The expression of *PfTOPOVIB* and *PfSPO11* was induced at the late schizont stage of the parasite, when the nuclear replication is reported to have ceased (13). To rule out the possibility that the cDNA preparation is not contaminated with the genomic DNA, we did the amplification of topoisomerases with each of the stage-specific mRNAs, which were pretreated with DNase but not with reverse transcriptase. We found no amplicons in PCR samples not pretreated with reverse transcriptase, confirming that the cDNA samples were devoid of genomic DNA. We did the qRT-PCR analysis with two independent batches of parasites and found that *PfTOPOVIB* and *PfSPO11* expression remained significantly lower than *PfTOPOII* and *PfGYRASE* expression at the ES stage (Fig. 2C). To confirm the expression pattern at the protein level, we isolated total parasite proteins from the ES, MS, and LS stages and probed them with PfTopoVIB or PfSpo11 antibodies. Western blot analysis showed the presence of the PfTopoVI subunits exclusively at the LS stage of the parasites (Fig. 2D). We isolated the proteins from three independent batches of synchronous ES, MS, and LS stage-specific parasites and performed Western blot analysis for each set; eventually, the band intensity of each blot was analyzed using ImageJ analysis. We normalized the band intensity of PfTopoVIB and PfSpo11 with the loading control actin and plotted the intensity (Fig. 2E). The result showed that the levels of PfSpo11 and PfTopoVIB expression were upregulated 5-fold and 10-fold, respectively, at the LS stage compared to the ES/MS stage. We also performed the indirect immunofluorescence assay to visualize the expression of PfTopoVI subunits in three distinct schizont stages of the parasite. Alexa Red 594-conjugated secondary antibody was used to visualize the red fluorescence for both PfTopoVIB and PfSpo11 by using Nicon Eclipse NiE AR fluorescence microscope. DAPI was used to stain the nucleus. We scanned hundreds of cells and found that both the subunits are predominantly expressed at the late schizont stage of the parasite. Hence, our study indicates that PfTopoVI may not have any function during genome replication; rather, it may play a role in genome segregation.

### Stage-specific promoter activity of PfTopoVI subunits.

**FIG 3:**
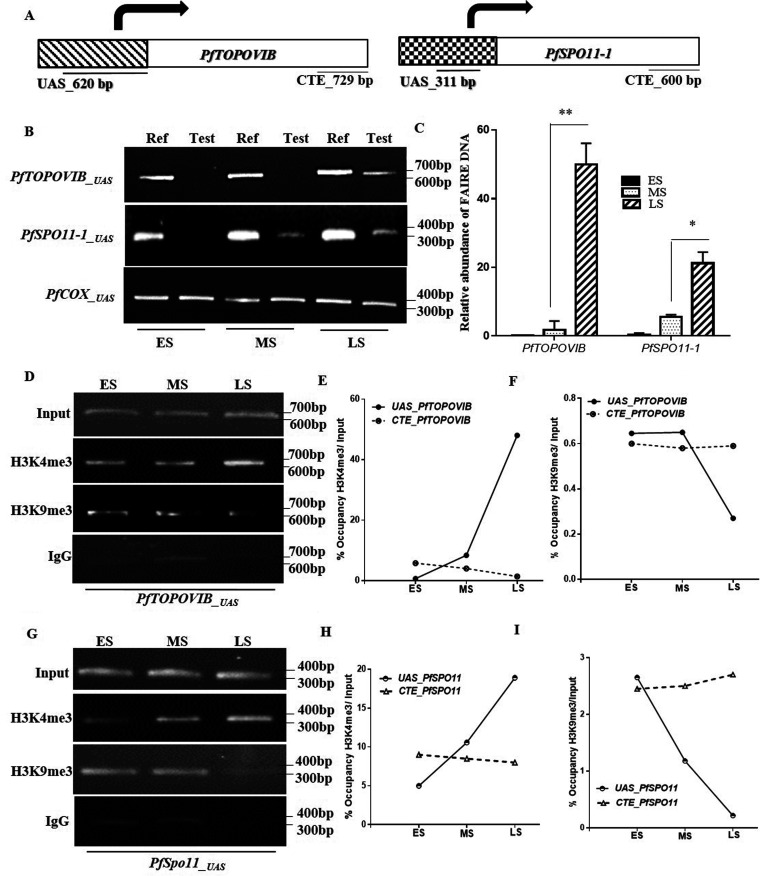
Stage-specific promoter activity of PfTopoVI subunits. (A) The positions of the upstream activator sequence (*620__UASPfTopoVIB_*) and C-terminal end (*729_CTE*) of *PfTOPOVIB* used in ChIP analysis are presented in the left panel. Similarly, the position of the UAS and CTE of *PfSPO11* (i.e., *311__UASPfSpo11_* and *600_CTE*) used in ChIP analysis are presented in the right panel. (B) FAIRE was performed with tightly synchronized ES, MS, and LS stages of the parasite to find that the *PfTOPOVIB__UAS_* and *PfSPO11__UAS_* chromatin remained loosely packed at the LS stage. *COX3* was used as a normalizing control. Lane 1, reference at ES; lane 2, test at ES; lane 3, reference at MS; lane 4, test at MS; lane 5, reference at LS; lane 6, test at LS. (C) The experiment described above was done with two independent sets of experiments, and the mean values ± SD were plotted, *P* values were calculated using two-tailed Student's *t* test (**, *P* < 0.01; *, *P* < 0.05). Both *PfTOPOVIB__UA_*_S_ and *PfSPO11__UAS_* are present as the nucleosome-free DNA at the LS stage. (D) ChIP assay was performed with the ES, MS, and LS stage-specific parasites using anti-H3K4me3 and anti-H3K9me3 antibodies to find their recruitment at the *PfTOPOVIB__UAS_*; IgG was used as a negative control. (E) qPCRs from two independent sets of ChIP assays were performed, and mean values ± SD were plotted. The recruitment of H3K4me3 to *UAS_PfTOPOVIB* was found to increase by 6-fold at the LS stage compared to that of the MS stage, although recruitment of H3K4me3 to *CTE_PfTOPOVIB* at the same stage was negligible. (F) The recruitment of H3K9me3 to *UAS_PfTOPOVIB* was found to decrease by 2-fold at the LS stage compared to that of the MS stage; however, recruitment of *CTE_PfTOPOVIB* under the same conditions remains unaltered. (G) The ChIP assay was performed with the ES, MS, and LS stage-specific parasites using anti-H3K4me3/anti-H3K9me3-specific antibodies to determine their recruitment at the *PfSPO11__UAS_*; IgG was used as a negative control. (H) qPCR using two independent sets of ChIP assays was performed, and mean values ± SD were plotted. The recruitment of the activation mark to *UAS_PfSPO11* was increased by 2-fold at the LS stage compared to that of the MS stage; however, recruitment under the same conditions at *CTE_PfSPO11* was not altered within the different stages. (I) The recruitment of the repressor mark showed a downward trend in *UAS_PfSPO11* and was almost negligible at the LS stage; however, the repressor mark remained bound at *CTE_PfSPO11* at all schizont stages.

To further validate the unique expression pattern of PfTopoVI subunits, we wanted to investigate the chromatin compaction of the promoter regions of *PfTOPOVIB* and *PfSPO11* at different developmental stages of the parasite. To this end, we used formaldehyde-assisted isolation of regulatory elements (FAIRE), which allows one to determine whether a specific region of chromatin is in nucleosome-free state or a nucleosome-bound state. Our assay was aimed at identifying whether the promoter regions of *PfTOPOVIB* and *PfSPO11* were indeed active specifically at the LS stage. We used 620-bp and 311-bp upstream activator sequences (UASs) from the translation start sites (ATG) of *PfTOPOVIB* and *PfSPO11*, respectively, for our analysis, as shown in Fig. 3A. As mitochondrial DNA (mtDNA) is not associated with the nucleosome, we used the *COX3* promoter sequence as the normalizing control. We found that the promoter of *PfTOPOVIB* remains in a nucleosome-bound state at the ES and the MS stages, whereas it is shifted to the nucleosome-free state at the LS stage of the parasite (Fig. 3B). The promoter of *PfSPO11* shows a similar pattern to *UAS*_*_PfTOPOVIB_*; however, it shows slightly loose chromatin compaction even in the mid-schizont stage. We repeated this experiment, and quantification of gel images revealed 25-fold and 4-fold relaxation in the chromatin compaction of the *PfTOPOVIB* and the *PfSPO11* promoters, respectively, at the LS stage of the parasite (Fig. 3C) compared to the MS stage. We determined the occupancy of two established epigenetic marks (14), namely, the activation mark H3K4me3 and the repression mark H3K9me3 to the promoter-proximal regions of *PfTOPOVIB* and *PfSPO11* at various schizont stages. For that, we performed chromatin immunoprecipitation (ChIP) analysis with the synchronous cultures of the ES, MS, and LS stages of the parasites using anti-H3K4me3, anti-H3K9me3, or anti-IgG antibodies. We found 6-fold and 2-fold enrichment of H3K4me3 at the promoter-proximal region of both *PfTOPOVIB* (Fig. 3D and E) and *PfSPO11-1* (Fig. 3G and H), respectively, especially at the LS stage. On the contrary, we found negligible recruitment of H3K9me3 at the *PfTOPOVIB_UAS_* and *PfSPO11-1_UAS_* at the ES stage, which was further decreased at the LS stage. To ascertain the specificity of the recruitment of H3K4me3 or H3K9me3 at the promoter-proximal region of *PfTOPOVIB/PfSPO11*, we performed the ChIP experiment with probes located within the 3′ end of the ORF of the aforementioned genes (C-terminal end [CTE] probes), as shown in Fig. 3A. However, the levels of recruitment of H3K4me3 at the CTE regions of *PfTOPOVIB* or *PfSPO11* were found be negligible, with no further increase at the LS stage (Fig. 3E and F and Fig. 3H and I). Similarly, the recruitment of H3K9me3 remained unchanged at the CTE region of *PfTOPOVIB* or *PfSPO11* within the different stages of schizont. Together, we conclude that the promoter-proximal sequences of *PfTOPOVIB* remain as heterochromatin during the ES/MS stage and undergo active transcription only at the LS stage. In case of *PfSPO11_UAS_*, we observe that the promoter-proximal region shows little activity in the MS stage and shows highest activity in the LS stage.

### PfTopoVIB and PfSpo11 form the functional holoenzyme in the parasite.

**FIG 4:**
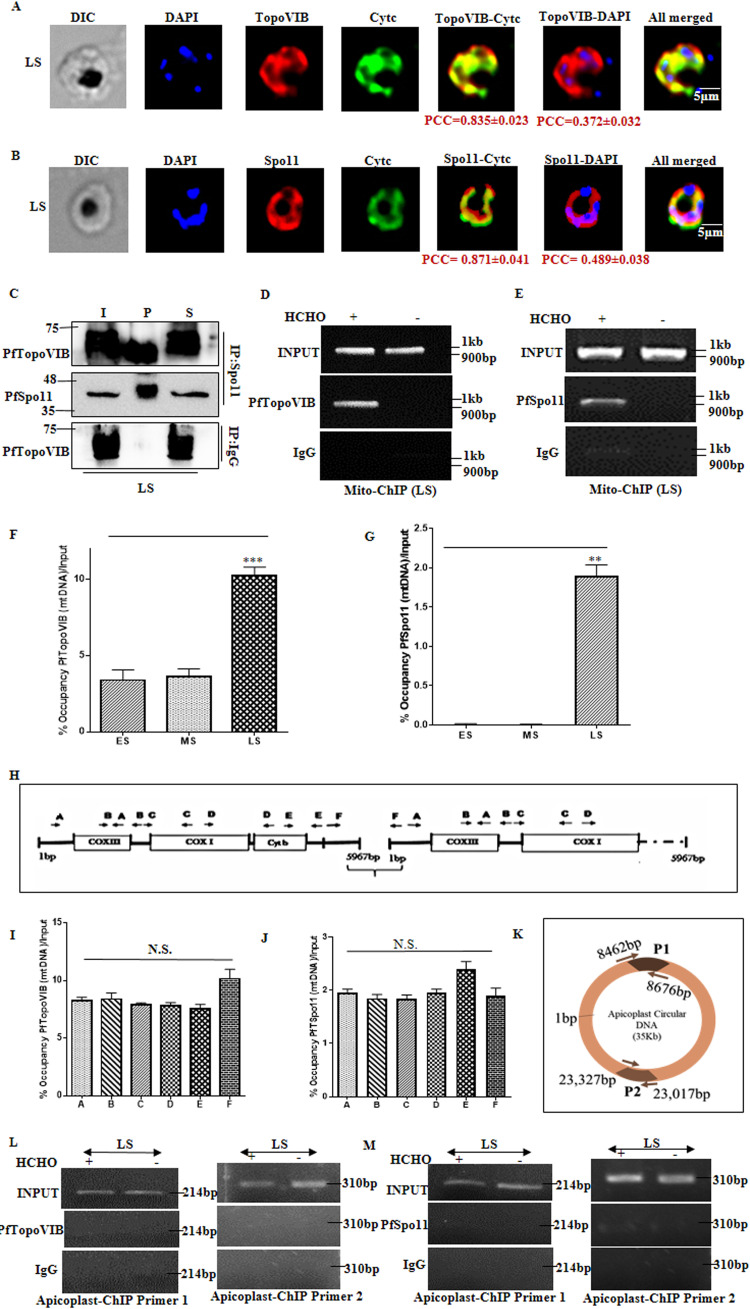
PfTopoVIB and PfSpo11 form the functional holoenzyme in the parasite. (A and B) Indirect immunofluorescence of LS stage-specific parasites shows the distribution of both PfTopoVIB (red) and PfSpo11 (red) in the parasite nucleus and mitochondria. After calculating the average (mean ± standard error of the mean [SEM]) PCC values (*n* = 25) in each case, PfTopoVIB and PfSpo11 fluorescence showed a moderate correlation (PCC < 0.5) with nuclear stain DAPI (blue) but strong correlation (PCC > 0.8) with Cytc (green). The average MOC for the fraction of red fluorescence of PfTopoVIB overlapping green Cytc was 0.964, and that for the fraction of PfSpo11 fluorescence overlapping green Cytc was 0.938. (C) Western blot analysis showing the coimmunoprecipitation of PfTopoVIB with PfSpo11 from the synchronized LS stage of the parasite lysate. Pulldown was done with anti-PfSpo11 and IgG antibodies. I, input; P, immunoprecipitation (IP) pellet fraction; S, supernatant. (D) Chromatin immunoprecipitation showing recruitment of PfTopoVIB on the mitochondrial genome at the LS stage. Samples without formaldehyde treatment were used as a negative control to confirm the specific recruitment. (E) Chromatin immunoprecipitation showing recruitment of PfSpo11 on the mitochondrial genome at the LS stage. Samples without formaldehyde treatment were used as a negative control to confirm the specific recruitment. (F and G) Graphical representation of relative occupancy (percentage of input) of PfTopoVIB and PfSpo11, respectively, on the mitochondrial genome in parasites at different developmental stages as quantified by qPCR: ES (35 to 36 hpi), MS (39 to 40 hpi), and LS (44 to 45 hpi). The experiment was done with three independent batches of parasites, and the mean values ± SD were plotted. *P* values were calculated using two-tailed Student's *t* test (***, *P* < 0.001; **, *P* < 0.01; N.S., not significant); the data were normalized using the respective IgG IP values. (H) Schematic representation of all the primers used in the ChIP assay, represented as A to F, each covering a 1-kb region of the mitochondrial genome. (I) PfTopoVIB shows uniform percentage of occupancy at different segments of mitochondrial genome (A to F) at the LS stage. (J) PfSpo11 also shows uniform percentage of occupancy at different segments of mitochondrial genome (A to F) at the LS stage. In both the cases in panels I and J, the relative occupancy was quantified by qPCR and the mean values ± SD were plotted. *P* values were calculated using two-tailed Student's *t* test (N.S., not significant); the data were normalized using respective IgG-IP values. (K) Schematic representation of the primers used in the apicoplast ChIP assay, represented as P1 and P2, each covering 214 bp and 310 bp, respectively, of the apicoplast genome; (L) chromatin immunoprecipitation showing no recruitment of PfTopoVIB on apicoplast genome (P1 and P2) at the LS stage; (M) chromatin immunoprecipitation showing no recruitment of PfSpo11 on apicoplast genome (P1 and P2) at the LS stage.

We determined the localization of PfTopoVI subunits within the parasite. For that, we harvested LS stage-specific parasites and performed indirect immunofluorescence to visualize PfTopoVIB and PfSpo11 as red fluorescence, as shown in Fig. 2F and G, respectively. We determined the average Pearson correlation coefficient (PCC) for 20 to 25 images to evaluate the localization of PfTopoVIB or PfSpo11 with the nuclear stain DAPI, as shown in Fig. 4A and B; the values are presented underneath the images in panels A and B. We used anti-cytochrome *c* (anti-Cytc) antibody to visualize the green fluorescence of mitochondrial protein Cytc, where the secondary antibody was conjugated with Alexa Fluor 488. We determined the average PCC to evaluate whether red signals specific to PfTopoVIB or PfSpo11 colocalize with the green fluorescence of Cytc; the average values are presented underneath the figure panels. We conclude that both the subunits are predominantly present in mitochondria as the average PCC values of each subunit show stronger correlation with Cytc (PCC > 0.8) and moderate correlation with DAPI (PCC < 0.5). We have provided additional cell images to show the localization of PfTopoVIB and PfSpo11 in the supplemental material (see Fig. S1C and D, respectively). Next, we determined the physical association between PfTopoVIB and PfSpo11 within the parasite by employing a coimmunoprecipitation assay. The LS stage-specific parasites were immunoprecipitated with PfSpo11-specific antibody, and the pellet fraction was probed with PfTopoVIB antibody. We found that PfTopoVIB was coprecipitated with PfSpo11, thus establishing a physical association between the two subunits (Fig. 4C). In a parallel experiment, immunoprecipitation was done with IgG, and when probed, no PfTopoVIB protein was detected in the pellet fraction. Thus, our study confirmed the presence of PfTopoVI holoenzyme at the late schizont stage of the parasite. As the holoenzyme expression does not correlate with the onset of replication of the parasite genome, we predict that it might function in mitochondrial genome segregation, which was earlier reported to occur at the late schizont stage of the parasite (4). In order to evaluate their possible function in the mitochondrial genome segregation, we determined whether the two subunits of PfTopoVI interact with the mitochondrial DNA. We employed the chromatin immunoprecipitation (ChIP) assay to detect the recruitment of PfTopoVIB and PfSpo11 to the mitochondrial genome in the presence or absence of formaldehyde cross-linking. We found the specific binding of PfTopoVIB (Fig. 4D) and PfSpo11 (Fig. 4E) to the mitochondrial genome in the LS stage-specific parasites and found that the mtDNA was only amplified in the presence of formaldehyde. We isolated synchronous ES (35 to 36 hpi), MS (39 to 40 hpi), and LS (44 to 45 hpi) stage-specific parasites and quantified the percentage of occupancy of PfTopoVIB of mtDNA with respect to the input by employing quantitative PCR (qPCR). We found that PfTopoVIB recruitment to the mtDNA is positively correlated with its expression and is significantly enriched at the LS stage of the parasites compared to the other stages (Fig. 4F). Similarly, ChIP was done with PfSpo11-specific antibody for the stage-specific parasites mentioned above. The qPCR showed that the percentage of occupancy of PfSpo11 in mtDNA was highest in the LS stage-specific parasites (Fig. 4G). To evaluate whether the enzyme shows any preference in the association toward any specific parts of mtDNA, we used a set of six primers (A to F), as presented schematically in Fig. 4H. Each primer pair results in 1-kb amplified fragments, and together A to E encompass the entire mitochondrial genome; additionally, the primer pair F has been designed such that it amplifies the junctional sequence and produces the amplicon from circular mtDNA or when two monomeric mtDNA units form concatemers. With the LS stage-specific PfTopoVIB-mtDNA as well as PfSpo11-mtDNA IP sample, we monitored the percentage of occupancy of the subunits in various parts (A to F) of mtDNA. The qPCR analysis showed no significant difference between the occupancies of PfTopoVIB (Fig. 4I) and PfSpo11 (Fig. 4J) of any specific regions of mtDNA. Furthermore, to evaluate whether the recruitment of the enzyme is specific to the mitochondrial genome or not, we monitored the association of both the subunits across the apicoplast genome by using two primer sets, P1 and P2, which cover 214 bp and 310 bp, respectively, as shown in Fig. 4K. In the LS stage-specific PfTopoVIB and PfSpo11 IP samples, we didn’t find any occupancy in the apicoplast genome (Fig. 4L and M). Together, these studies indicate a probable functional association of PfTopoVI during mitochondrial genome segregation at the late schizont stage of the parasite.

### PfTopoVIB inhibitor radicicol and atovaquone potentiate each other.

Since atovaquone collapses the mitochondrial membrane potential of the parasite (15), we speculated that treatment with it should reduce the mitochondrial import of PfTopoVI subunits. On the other hand, since PfTopoVI is involved in mitochondrial genome maintenance, inhibition of this enzyme complex by radicicol (5) should affect the effective replication of mitochondrial genome. In our earlier work, it was observed that radicicol treatment indeed reduced the mitochondrial genome content of the parasite (9); hence, we speculate that the total amount of transcripts of mitochondrial genes, including cytochrome *b* and subunit I of cytochrome *c* oxidase (Cox I), which are the targets of atovaquone (*bc*_1_ complex), will be decreased. Hence, we hypothesize that atovaquone and radicicol should potentiate each other’s action. We treated the synchronous trophozoite stage-specific 3D7 parasites with various doses of atovaquone for 48 h and measured the parasite survivability by the SYBR green method. The 50% inhibitory concentration (IC_50_) value obtained was 1.4 nM under our experimental condition (Table 1; Fig. S1A). When a similar experiment was performed in the presence of an IC_50_ of radicicol of 8.05 μM (Fig. S1B) (9), we observed a significant shift in the IC_50_ of atovaquone, and it was reduced to 0.12 nM (Table 1). Thus, radicicol imparted 11.7-fold potentiation to atovaquone (Table 1). Similarly, we observed that the presence of an IC_50_ of atovaquone reduces the IC_50_ of radicicol to 1.7 μM. Thus, atovaquone was also found to potentiate radicicol by 4.7-fold (Table 1).

**TABLE 1:** IC_50_ for strain 3D7 of radicicol in combination with atovaquone and vice versa

Drug or drug combination	IC_50_	Potentiation factor
Radicicol alone	8 μM	1
Radicicol + atovaquone^*a*^	1.7 μM	4.7
Atovaquone alone	1.4 nM	1
Atovaquone + radicicol^*b*^	0.12 nM	11.67

a The IC_50_ of atovaquone in strain 3D7 was used.

b The IC_50_ of radicicol in strain 3D7 was used.

### The PfTopoVIB inhibitor radicicol interacts with atovaquone in a synergistic manner.

**FIG 5:**
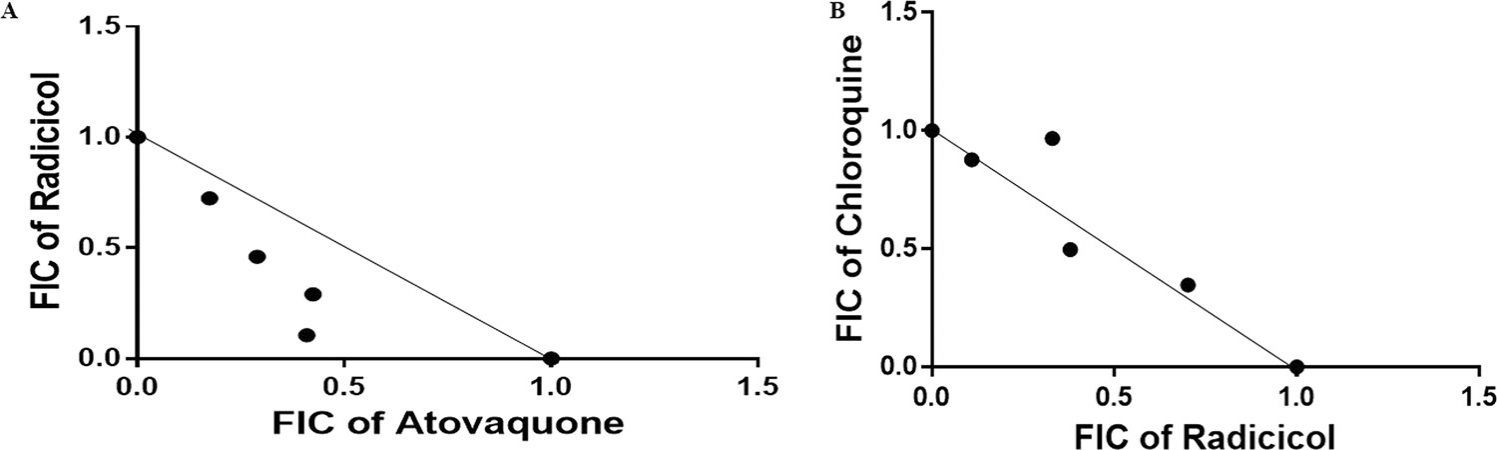
PfTopoVIB inhibitor radicicol and atovaquone show synergism with each other. (A) Synergistic interaction between the PfTopoVIB inhibitor radicicol and atovaquone was determined by plotting an isobologram of radicicol and atovaquone interaction in the 3D7 strain. Each point represents the mean half-maximal inhibitory concentration (IC_50_) of the drug combination from three independent experiments. A solid line was drawn between the IC_50_ values of each of the drugs radicicol and atovaquone when used alone. FIC, fractional inhibitory concentration. (B) In a similar way, an isobologram of radicicol and chloroquine interaction in the 3D7 strain was plotted. Each point represents the mean IC_50_ of the drug combination experiment from two independent experiments. A solid line was drawn between the IC_50_ values of each drug when used alone.

We studied whether the interactions between the PfTopoVI inhibitor radicicol and atovaquone are synergistic or additive. For that, we performed a fixed-ratio drug combination assay. For each combination of the drugs, the dose-response curves were plotted (data not shown) and the fractional inhibitory concentration (FIC) was calculated and tabulated (Table 2). Subsequently, the sum of FIC values was calculated (Table 2) and plotted in an isobologram (Fig. 5A). The isobologram shows that the interaction between radicicol and atovaquone is synergistic in nature. In order to investigate the specificity of radicicol-atovaquone interaction, we used an unrelated drug, chloroquine, and determined its interaction with radicicol. We calculated the FIC and ΣFIC (Table 2), and the isobologram (Fig. 5B) was plotted. We found that a ΣFIC of ≥1 represents no interaction between radicicol and chloroquine. Thus, the synergistic interaction between PfTopoVI inhibitor and chemical that collapses mitochondrial membrane potential provides supporting evidence that PfTopoVI is a mitochondrial topoisomerase.

**TABLE 2:** FIC values for strain 3D7 for the combinations of atovaquone and radicicol and radicicol and chloroquine

Drug ratio	FIC of:		ΣFIC
	Atovaquone or radicicol	Radicicol or chloroquine	
Atovaquone/radicicol	Atovaquone	Radicicol	
5:0	1	0	1
4:1	0.41	0.106	0.5
3:2	0.425	0.291	0.716
2:3	0.29	0.460	0.75
1:4	0.175	0.724	0.899
0:5	0	1	1
Radicicol/chloroquine	Radicicol	Chloroquine	
5:0	1	0	1
4:1	0.702	0.347	1.049
3:2	0.38	0.497	0.877
2:3	0.33	0.967	1.297
1:4	0.11	0.877	0.987
0:5	0	1	1

### Atovaquone reduces mitochondrial import and mtDNA recruitment of PfTopoVIB and PfSpo11 in a dose-dependent manner.

**FIG 6:**
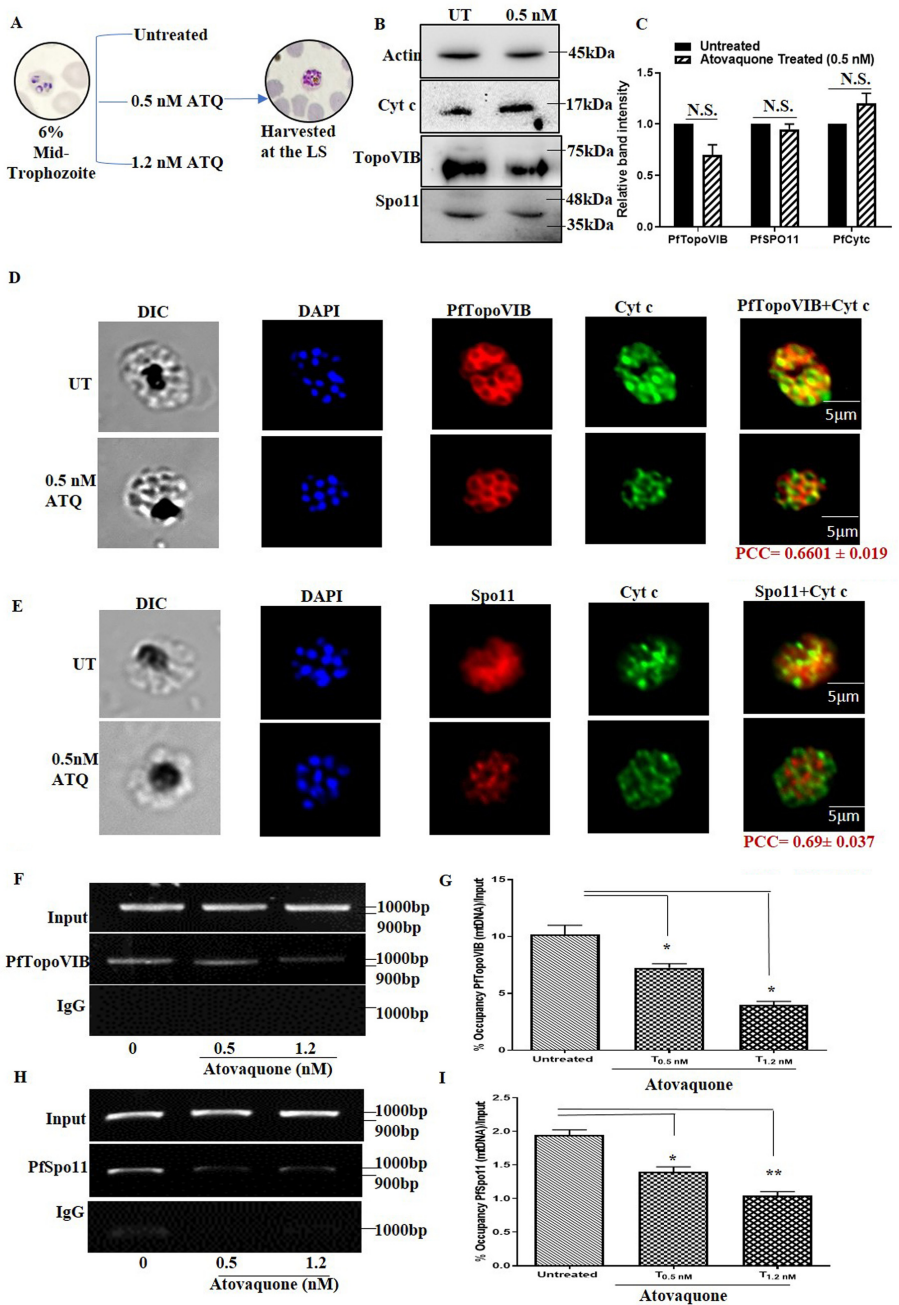
Atovaquone reduces mitochondrial import and mtDNA recruitment of PfTopoVIB and PfSpo11 in a dose-dependent manner. (A) Schematic presentation of the assay in the presence of atovaquone. Synchronized mid-trophozoite-specific parasite cultures (6% parasitemia) were divided into 3 parts: one set was untreated, and the other two sets were treated with different doses of atovaquone. Subsequently parasites were harvested at the LS stage (44 to 45 hpi); parasites treated with 0.5 nM atovaquone were subjected to IFA. The ChIP assay was performed with parasites treated with 0.5 and 1.2 nM atovaquone along with the untreated control. (B) Western blot analysis showed no significant difference in the endogenous levels of PfSpo11, PfTopoVIB, and PfCytc in the atovaquone-treated culture compared to the untreated condition. (C) Graph displayed the relative band intensities of PfTopoVIB, PfSpo11, and PfCytc, from the Western blots with two independent protein preparations and calculated using ImageJ software. (D and E) Indirect immunofluorescence assay showed that there was a significant reduction in the colocalization (yellow) of Cytc (green) and PfTopoVIB/PfSpo11 (red), respectively, in the parasites treated with 0.5 nM atovaquone compared to the untreated parasites. The mean PCC values were determined from 25 individual cells from each of the IFA, and the average value is presented at the bottom of merged image. (F) The recruitment of PfTopoVIB on the mitochondrial genome was significantly decreased in the presence of atovaquone in a dose-dependent manner. (G) Quantitation of PfTopoVIB recruitment (percentage of occupancy/input) was calculated by real-time PCR analysis with two independent sets of experiments, and the data were presented. (H) Recruitment of PfSpo11 on the mitochondrial genome was also decreased with increasing concentrations of atovaquone. (I) Quantitation of PfSpo11 recruitment (percentage of occupancy/input) was calculated by real-time PCR analysis with two independent sets of experiments, and the data were presented. *P* values were calculated using a two-tailed Student's *t* test (**, *P* < 0.01; *, *P* < 0.05; N.S., not significant).

In order to support our conclusion further, we sought to determine whether atovaquone treatment really affects the mitochondrial import and the mitochondrial DNA recruitment of PfTopoVI. To that end, the synchronous mid-trophozoite-specific parasite culture was treated with increasing doses of atovaquone and allowed to grow until the parasites reached the late schizont stage. The plan of the experiment is schematically presented in Fig. 6A. Subsequently, the cultures were harvested and the mitochondrial localizations of PfTopoVIB and PfSpo11 were measured using an immunofluorescence assay (IFA) and compared with those of the untreated parasites. In order to rule out the possibility that atovaquone treatment reduces the overall expression of Cytc or PfTopoVI, we performed Western blot analysis under the treatment condition using 0.5 nM atovaquone and compared the result with that from the untreated sample (Fig. 6B). The experiment was repeated with two independent batches of cells, and we calculated the band intensity of each of the PfTopoVI subunits in the Western blots using ImageJ and plotted the intensities (Fig. 6C). We found no significant difference in the levels of expression of PfTopoVIB, PfSpo11, and PfCytc under the treated condition. We calculated the average PCC values from 25 individual cells that were treated with 0.5 nM atovaquone and compared them with those of the untreated parasites. We observed that in the parasites treated with 0.5 nM atovaquone, the average PCC values were shifted from 0.835 to 0.66 in the case of PfTopoVIB (Fig. 6D), and the same values were shifted from 0.871 to 0.69 in the case of PfSpo11 (Fig. 6E). We conclude that the degree of colocalization between PfTopoVI with Cytc decreased in atovaquone-treated parasites. To validate this further, we determined the recruitment of these two subunits to the mtDNA under the atovaquone-treated condition. The ChIP assay with PfTopoVIB antibody was done with atovaquone concentrations of 0.5 nM and 1.2 nM, and there was a gradual decrease in recruitment of PfTopoVIB to the mitochondrial genome (Fig. 6F). The occupancy of PfTopoVIB with respect to the input DNA was quantified by real-time qPCR and is presented graphically in Fig. 6G. We observed about 30% reduction in the occupancy of PfTopoVIB in the mitochondrial genome in the presence of the sublethal doses (0.5 nM) of atovaquone, which showed a further reduction at a higher concentration of atovaquone. Similarly, we investigated the mitochondrial recruitment of PfSpo11 at three doses of atovaquone as described above. We observed a similar effect (Fig. 6H), and real-time quantification analysis revealed a dose-dependent reduction of recruitment of PfSpo11 to the mitochondrial genome (Fig. 6I).

### PfTopoVIB and HsTopoVIBL differ in their Bergerat folds.

**FIG 7:**
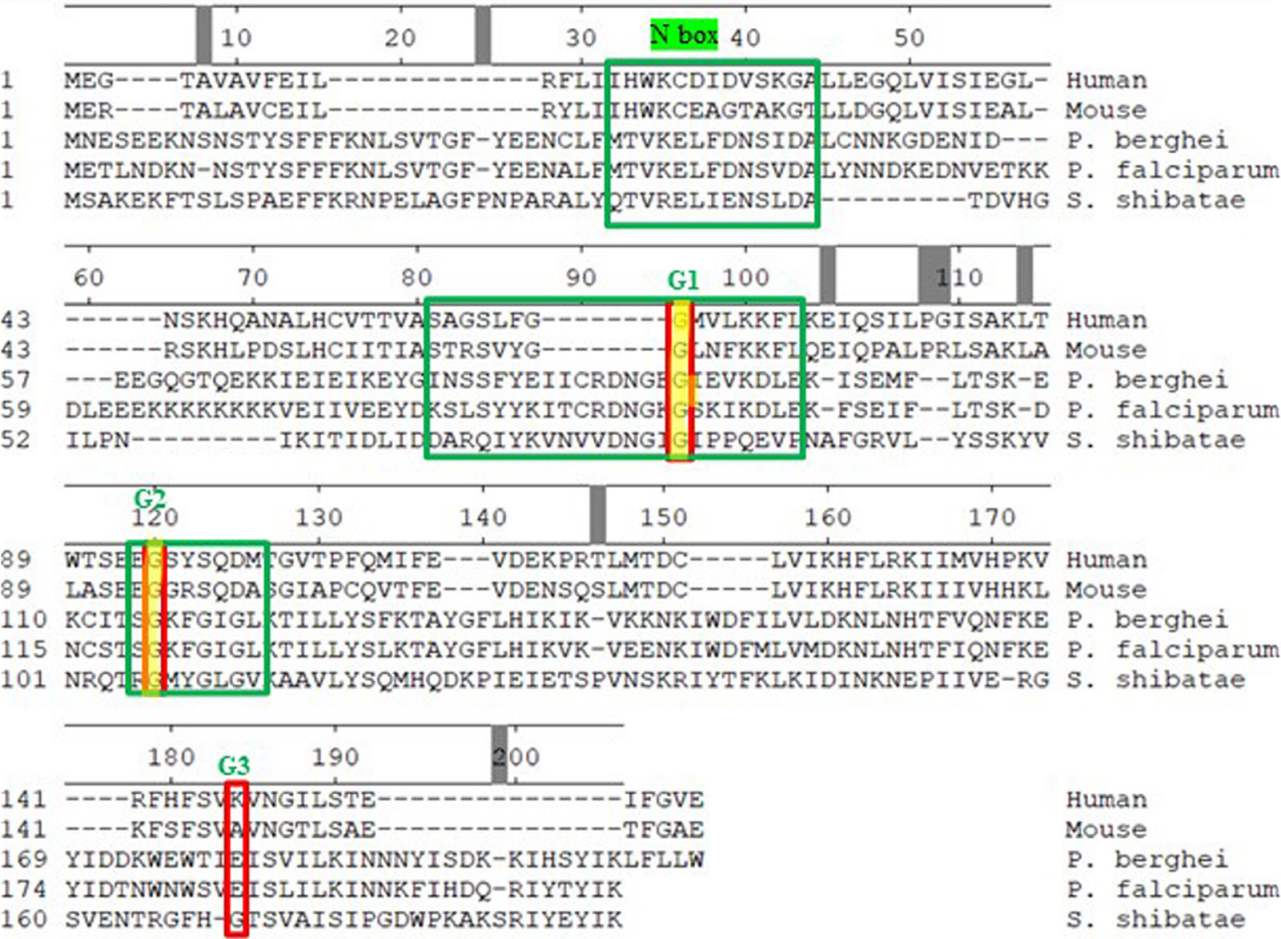
Multiple-sequence alignment of TopoVIB/TopoVIBL proteins from *S. shibatae* (Ss), P. falciparum (Pf), P. berghei (Pb), *M. musculus* (Mm), and H. sapiens (Hs). The UniProt ID numbers and the residue numbers considered in the alignment are given next to the species. The conserved glycines are shown in red boxes, and the four core elements of the Bergerat fold are shown in blue boxes.

**FIG 8:**
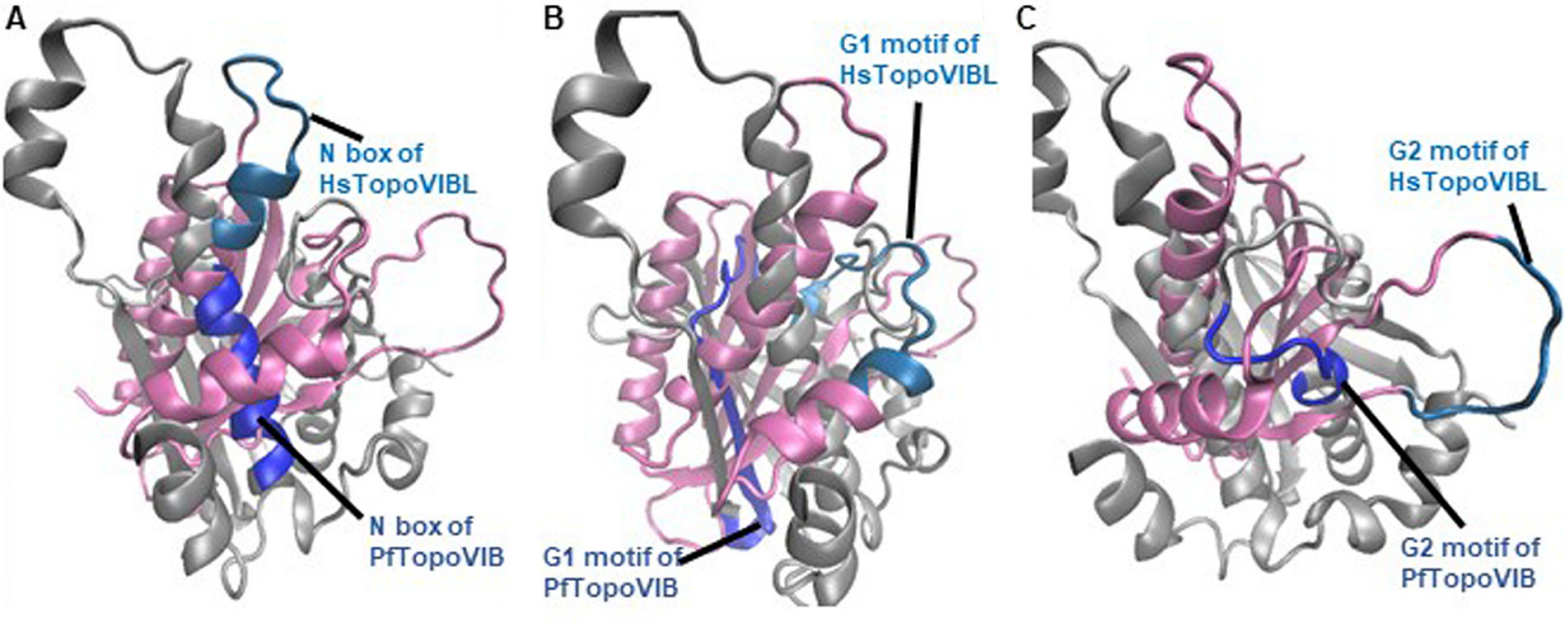
Structural alignment of the Bergerat fold region of PfTopoVIB (shown in silver) and HsTopoVIBL (shown in pink) shown from different angles: The core elements of the Bergerat fold are shown in blue. The difference in spatial orientation of these core elements can be seen in the figure. (A) N box; (B) G1 motif; (C) G2 motif.

There is only 10% identity in the amino acid sequences between *Plasmodium* TopoVIB and human TopoVIBL protein (2). A multiple-sequence alignment of the Bergerat fold region from different species is shown in Fig. 7. Although there are a number conserved residues in the N box within Sulfolobus shibatae and *Plasmodium* species, there is a low degree of conservation between human/mouse and *Plasmodium* within the signature box N, G1, and G2 motifs, apart from the conserved glycines. The glycines in the G3 motif are not conserved though. A structural alignment of the Bergerat fold region from PfTopoVIB and HsTopoVIBL is shown in Fig. 8. The structures of the two proteins differ significantly in the Bergerat fold region. This suggests that there could be major differences in the catalytic activity of the two proteins.

## DISCUSSION

This study demonstrates the existence of *Plasmodium* topoisomerase VI holoenzyme complex in the parasite lysate at the LS stage of the parasite. Furthermore, in this article we have established topoisomerase VI as a mitochondrial topoisomerase of the parasite. First, we have shown the localization of a topoisomerase holoenzyme (PfTopoVIB and PfSpo11) within the mitochondria. Second, both subunits remain associated with the whole mitochondrial genome in accordance with their expression. Third, the disrupter of mitochondrial membrane potential atovaquone inhibits the mitochondrial import and the recruitment of PfTopoVI subunits to the mitochondria in a dose-dependent manner. Finally, atovaquone and the PfTopoVIB inhibitor radicicol potentiate each other’s action and display synergistic interaction with each other.

It was proposed earlier that the *Plasmodium* mitochondrion undergoes a rolling circle mode of replication, and during replication there is homologous recombination between circles and the termini of the linear molecules, which generates complex lariat-like structures (16, 17). This necessitates the involvement of a type II topoisomerase, which should decatenate the replicated mitochondrial genome before endoreduplication so that it can be segregated to the progeny. It was reported earlier that PfGyrase, another type II topoisomerase of the malaria parasite, was a bona fide apicoplast-specific topoisomerase (18) and was not detected in mitochondria. Our study suggests a likely role of PfTopoVI in the segregation of the mitochondrial genome. We found that the expression of PfTopoVI is very tightly regulated, and both the subunits are induced during the late schizont (44 to 45 hpi) stage of the parasite, before the initiation of genome segregation. Furthermore, the subunits’ recruitment to mitochondrial genome correlates well with their expression in the parasite. It can be speculated that inhibition of PfTopoVI might impair the endoreduplication due to the inhibition of mitochondrial genome segregation. Indeed, this notion is supported by our earlier observations that radicicol (PfTopoVIB inhibitor) treatment arrests the parasites at the late schizont stage and inhibits the transition from schizont to ring stage (9).

We do not rule out the possibility that PfTopoVI has an additional nuclear function as the PCC values determined through our experiments indicate a moderate correlation between PfTopoVIB/PfSpo11 and DAPI. However, in the present work we didn’t explore the nuclear function of PfTopoVI, if any.

The other type II topoisomerase transcripts were detected in most stages, including the LS stage of the parasite. It is likely that these topoisomerases could be involved in various cellular function involving DNA transactions at all the stages. Alternatively, it could be possible that the transcript of other topoisomerases is very stable, having a longer half-life, and hence they are detected even at the LS stage.

*Plasmodium* topoisomerase VI is a type IIB topoisomerase, which can be exploited as a novel antimalarial target due to its absence in humans. The cross-reactivity of human Spo11 antibody with PfSpo11 indicates that there could be structural conservation between human and *Plasmodium* Spo11. On the contrary, there is much less sequence identity (10%) between PfTopoVIB and mouse/human TopoVIBL protein. Predicted structures of these two subunits show that the ATP binding pocket of PfTopoVIB, namely, the Bergerat fold, does not superimpose with the similar fold present in human TopoVIBL protein. Thus, a specific inhibitor that binds to the ATP binding pocket of PfTopoVIB is less likely to block the function of the TopoVIBL protein of human. Our earlier work demonstrated that radicicol inhibits the decatenation activity of PfTopoVI. Radicicol is a pan-inhibitor of heat shock protein Hsp90 and can bind to the Bergerat fold of the human heat shock protein Hsp90; however, apart from this Bergerat fold, TopoVIB and Hsp90 are very different molecules, and hence it could be possible to identify specific inhibitors of PfTopoVIB that do not inhibit human Hsp90. Future studies are required to screen small molecule inhibitors that will specifically block PfTopoVIB and not human Hsp90 (19). Any inhibitor specifically targeting the PfTopoVIB subunit will inhibit the function of the entire enzyme complex PfSpo11-TopoVIB, and would arrest the endoreduplication of the parasite.

## MATERIALS AND METHODS

### Generation of plasmids and yeast strains.

**(i) Plasmids.** Full-length *ScSPO11* was cloned in the yeast expression vector *pRS313* by amplifying yeast genomic DNA using the forward primer OSB 643 (5′ GAC GGA TCC ATG GCT TTG GAG GGA TTG CG 3′) with a BamHI site and the reverse primer OSB 644 (5′ GAC GTC GAC TCA TTT GTA TTC AAA AAT TCT GGC 3′) with a SalI restriction site. The full-length *PfSPO11* was cloned in the *pRS313* vector by nondirectional cloning at the BamHI site, using the 3D7 cDNA as a template and primer pairs OSB 645 (5′ GAC GGA TCC ATG CCT CGT CTG GAT ATC 3′) and OSB 646 (5′ GAC GGA TCC TTA TAA AAG CTC CTT AAT GCG 3′), each having a BamHI restriction site. The *PfSpo11Y65F* mutant was also cloned in the *pRS313* vector at the BamHI site.

**(ii) Site-directed mutagenesis.** Point mutation (Y to F) was generated in *PfSPO11* by mutating the codon TAC to TTT using the splicing by overlap extension (SOE) PCR technique. To insert the point mutation at *PfSpo11Y65F*, the coding sequence was amplified in two segments. The first segment was amplified by using the primer pair OSB 645 (mentioned above) and OSB 652 (5′ TAT AAA TAA TTT TGG ATT GGT AAA AAA TAT TTG TC 3′); the second segment was amplified using the primer pair OSB 653 (5′ CAA CTT TAA GAC AAA TAT TTT TTA CCA ATC C 3′) and OSB 646 (described above). Subsequently, the full-length *PfSPO11* containing the Y65F mutation was amplified using the two segments as a template and primer pair OSB 645 and OSB 646 and cloned into the *pRS313* vector. The generation of mutation was confirmed by DNA sequencing.

**(iii) Yeast strains.** Empty *pRS313* vector, *pRS313-PfSPO11*, *pRS313-Pfspo11Y65F*, and *pRS313-ScSPO11* were transformed in a diploid *spo11Δ* BY4743 strain (MAT**a**/MATα *his3*Δ*1*/*his3*Δ*1 leu2*Δ*0*/*leu2*Δ*0 LYS2*/*lys2*Δ*0 met15*Δ*0*/*MET15 ura3*Δ*0*/*ura3*Δ*0 YHL022c*/*YHL022c*::*kanMX4*) to generate the strains PSY4, PSY1, PSY2, and PSY3, respectively.

### Yeast sporulation.

Each strain was inoculated in the histidine dropout medium and allowed to grow overnight at 30°C. The next morning, the cells were inoculated in presporulation medium (1% potassium acetate, 1% yeast extract, 2% peptone, 0.003% uracil, and 0.005% leucine) and further allowed to grow for 18 h at 30°C in a shaker incubator. After 18 h, when the optical density at 600 nm (OD_600_) reached 0.5, cells were washed 1 to 2 times with sterile Milli-Q water, further resuspended in the sporulation medium (1% potassium acetate, 0.003% uracil, 0.005% leucine), and allowed to grow in a shaking water bath at 18°C for 48 h. At the end of 48 h, cells were stained with DAPI and sporulation efficiency was calculated. We analyzed a total of 1,000 cells from each strain (by doing three independent sets of experiments) using fluorescence microscopy to count the number of cells that can form asci (3 or 4 spores). Subsequently, the sporulation efficiency was calculated using the following formula: % of sporulation = (cells containing 3 or 4 asci/total no. of diploid cells counted) × 100.

The mean values ± standard deviation (SD) were plotted for each strain using GraphPad Prism 6. The results represent mean ± SD. *P* values were calculated using the two-tailed Student's *t* test.

### Plasmodium falciparum culture.

P. falciparum 3D7 parasites were maintained with 5% hematocrit in RPMI 1640 medium (Himedia) supplemented with 0.5% (wt/vol) Albumax (Thermo Fisher Scientific) and 0.005% (vol/vol) hypoxanthine (Sigma). Parasites were maintained at 37°C using the candle jar method (20). Parasite growth was monitored by microscopic examination of Giemsa-stained slides. Cultured parasites were synchronized at the ring stage by treatment with 5% sorbitol (Sigma, St. Louis, MO, USA) as discussed earlier (21). We harvested the synchronized parasites that were grown for 35 to 36 hpi as early schizont (ES), 39 to 40 hpi as mid-schizont (MS), and 44 to 45 hpi as late schizont (LS). Each of the ES, MS, and LS stage-specific Giemsa-stained cultures was also confirmed according to its morphology under the microscope.

### Western blot analysis.

ES, MS, and LS stage-specific cultures were harvested at 5% parasitemia, and parasite protein was isolated. Western blot analysis was performed as described earlier (21). The blot was probed with rabbit anti-TopoVIB antibody (5) and rabbit anti-human Spo11 (anti-hSpo11) antibody (Invitrogen) at 1:500 and 1:3,000 dilutions, respectively. In order to detect PfSpo11 or ScSpo11, we used anti-human Spo11 antibody. Mouse anti-human actin 1 antibody (Abcam) was used as a normalizing control. Horseradish peroxidase (HRP)-conjugated anti-rabbit IgG (Promega) and anti-mouse (Santa Cruz Biotechnology) were used as the secondary antibodies in a 1:10,000 dilution. To probe the protein level in the presence of atovaquone, cytochrome *c* (Cytc) was used as a mitochondrial protein marker. For that, atovaquone-treated parasite proteins were probed with anti-mouse anti-Cytc (Abcam) at 1:3,000 dilutions. The Western blots were developed by using a chemiluminescence detection system (Pierce). Each experiment was repeated with three independent batches of cells, and band intensities were quantified using ImageJ software. Mean relative densities were plotted using GraphPad prism.

### RNA isolation and real-time analysis.

Total RNA was isolated from synchronized ES, MS, and LS stages of the 3D7 parasite as previously described (21). It was subjected to DNase I treatment (Fermentas) to eliminate DNA contamination. PCR without reverse transcriptase pretreatment was performed to confirm the absence of genomic DNA. cDNA was synthesized by using 1 μg of total RNA by using reverse transcriptase (Qiagen), and the cDNA product was then subjected to amplification using small *ARP* gene-specific primers OSB 94 and OSB 95 (21). *PfSPO11* expression was studied by amplifying the cDNA with gene-specific primers OSB 589 (5′ TGA TAT GTC CAT CGA GAA TCT TC 3′) and OSB 590 (5’CCT TAA TGC GAT TAT TTA TAT GTT C 3′). *PfTOPOVIB* (PF3D7_1365600) was amplified using the gene-specific primers OSB 548 (5′ GGT GTT CAG TTA GCA TCT TC 3′) and OSB 549 (5′ CAT TCA TCT TCA CCT TCA CC 3′). OSB 578 (5′ AAA CCA AGA TTA ACC TTA TCT G 3′) and OSB 579 (5′ TTA AAT GTT GTA TGA ACT ATC AC 3′) were used to amplify *PfTOPOII* (PF3D7_1433500). OSB 580 (5′ GGA AAA GGA CAT AGA ATC ATG 3′) and OSB 581 (5′ TCA GAT TAT GTC AAA ATA AAC C 3′) were used to amplify *PfGYRA* (PF3D7_1223300), and OSB 582 (5′ GTG AAT GAA GAG GGT TCG AC 3′) and OSB 583 (5′ CTG ATA ATG AAT TTG TAT TTT CC 3′) were used to amplify *PfGYRB* (PF3D7_1239500). For the real-time analysis, cDNA was diluted in a 1:50 ratio and used for PCR using a TaKaRa RT-PCR kit. Real time analysis was conducted using the Applied Biosystems 7500 Fast real-time PCR system. A threshold cycle (*C_T_*) value of *ARP* transcript was used as the normalizing control for the *C_T_* values of other *TOPOII* transcripts to obtain Δ*C_T_* values for each. The relative mRNA levels were gathered from the formula (change in mRNA level [2^Δ^*^CT^*]). The mean values ± SD were plotted using GraphPad Prism 6 software from two independent repeats.

### Chromatin immunoprecipitation assay.

Synchronized ES, MS, and LS stage-specific 3D7 parasites were harvested, each with 7 to 8% parasitemia, and we followed the standard procedure as described previously (21). Briefly, formaldehyde (37%) was added to the parasite culture so that its final concentration reached 0.5%, and then the culture was incubated at 37°C for 10 min. Subsequently, sonication (Elma; model-S-60H) was performed according to the standardized protocol (21) to generate small chromatin fragments. Protein-DNA complexes were then selectively immunoprecipitated using anti-PfTopoVIB (9) and anti-PfSpo11 antibodies (Invitrogen). Reverse cross-linking was performed using 5 M NaCl, and finally, DNA was extracted using proteinase K-phenol chloroform treatment. Recruitment of PfTopoVIB and PfSpo11 to mtDNA was quantified utilizing specific primer pairs covering different 1-kb regions (A to F) of the Plasmodium falciparum mitochondrial genome, as described earlier (21). Rabbit IgG was used as a control for the ChIP assay. For quantification of the mitochondrial association, real-time qPCR analysis was performed to amplify regions A to F utilizing the following primer pairs: A, OMKB 540 (5′ GAG TGG ATT AAA TGC CCA GCC 3′) and OSB 596 (5′ CAT TGG AAT GAG AGT TCA CCG 3′); B, OSB 493 (5′ TAC TCT GTA GTT TGT AGA GAT GC 3′) and OSB 597 (5′ TGT ATT TTC ATC TTT AAC TTC TGG 3′); C, OMKB 615 (5′ CTG GCC TAC ACT ATA AGA ACG 3′) and OSB 598 (5′ TGA AGA ATA TAA TTC AGT ACG TAG 3′); D, OSB 599 (5′ TAC TGG TTT AGA AGT TGA TAC TAG 3′) and OSB 600 (5′ATC TTG AAA TGC ACT TAC AGT TG 3′); E, OSB 601 (5′ TTA TCC TCT ATT CCA GTA GCA G 3′) and OSB 602 (5′ ACG ATA GCA TTA TCA GGA TGT G 3′); and F, OMKB 620 (5′ CGC TGA CTT CCT GGC TAA AC 3′) and OSB 603 (5′ GAA TTG AAG TGT GGA GAG AAT C 3′). To detect the recruitment of PfTopoVIB and PfSpo11 under the atovaquone-treated condition, tightly synchronized mid-trophozoite-treated parasite cultures (7 to 8% parasitemia) were treated with different doses of atovaquone (i.e., 0, 0.5, 1.2, and 2.4 nM) and allowed to grow until they reached the LS stage. Furthermore, LS stage-specific cultures were harvested in a similar manner as described above, and then ChIP was performed. To check the recruitment of H3K4me3 and H3K9me3 on *UAS_PfSPO11* and *UAS_PfTOPOVIB*, ChIP was performed with the ES, MS, and LS stages of the parasites. We determined the recruitment of both the subunits toward its C-terminal end (CTE), as shown schematically in Fig. 3A, which acts as a negative control in our assay. We used the antibodies against H3K4me3 (Millipore) and H3K9me3 (Millipore). Quantification of the recruitment was done using primer pairs OSB 463 (5′ AGC GGT ACC GTG GCA CCT TGT ATG TTT AC 3′) and OSB 464 (5′ AGC ATG CAT TAT TAT ACA CAA CAT AAA TAT ATA TA 3′) on *PfTOPOVIB_UAS_* and OSB 563 (5′ TTC CCC TAG TGT TAC ATT TGG 3′) and OSB 564 (5′ TAG GAA ATC ATA TTT TCA TTT TTA C 3′) on *PfSPO11_UAS_*. To check the recruitment of activation and repression mark to *CTE*_*PfTOPOVIB* and *CTE_PfSPO11*, we used the primer pairs OSB 548 and OSB 549 and OSB 589 and OSB 590, respectively. Rabbit IgG was used as a negative control.

### Formaldehyde-assisted isolation of regulatory elements.

The FAIRE (formaldehyde-assisted isolation of regulatory elements) assay was performed according to the previously published protocol (22). Briefly, synchronized parasite culture was divided into two parts (one as a reference and one as a test sample) and centrifuged at 3,000 rpm for 10 min. Briefly, 37% formaldehyde was added (final concentration of 1%) to the test culture, and the culture was incubated at 25°C at 80 rpm in an orbital shaker for 20 min. Glycine was added to a final concentration of 125 mM for 10 min at the same temperature to quench the formaldehyde. Cells were rinsed with phosphate-buffered saline (PBS) containing phenylmethylsulfonyl fluoride (Sigma), and the culture was then rinsed two more times. The cells were spun at 3,500 rpm for 15 min and frozen using liquid nitrogen. Cells were resuspended in 1 mL of lysis buffer (2% Triton X-100, 1% SDS, 100 mM NaCl, 10 mM Tris-HCl [pH 8.0], 1 mM EDTA) per 0.4 g of cells and incubated for 1 h for both reference and test samples. Samples were then sonicated for six sessions of 10-s bursts, followed by 5 min on ice. Cells were then spun at 16,000 × *g* for 20 min at 4°C. The supernatant obtained was subjected to phenol chloroform isoamyl alcohol (PCIA) treatment. After PCIA treatment, the aqueous layer was transferred into a new tube and precipitated using 2 volumes of 100% ethanol and 1/10 volume of sodium acetate. PCR analysis was performed using *PfTOPOVIB* and *PfSPO11* promoter-specific primer pairs OSB 463 and OSB 464 and OSB 563 and OSB 564, respectively (as mentioned above). *PfCOX* promoter-specific primers OSB 177 and OMKB 418 (22) were used as normalizing controls.

### Coimmunoprecipitation.

Coimmunoprecipitation (co-IP) was performed using the Pierce cross-link immunoprecipitation kit (Thermo Scientific) according to the manufacturer’s protocol. Briefly, the anti-PfSpo11antibody was coupled to Pierce protein A/G Plus agarose beads (provided in the kit). Cross-linking of the beads and antibody was done using disuccinimidyl suberate (DSS) solution. Furthermore, washing of the beads was performed as per the protocol to quench the cross-linking. Tightly synchronized LS stage parasites were harvested using 0.15% saponin treatment and were lysed using the immunoprecipitation (IP) lysis buffer (0.025 M Tris, 0.15 M NaCl, 0.001 M EDTA, 1% NP-40 and 5% glycerol [pH 7.4]). Centrifugation was performed to separate lysate from the cell debris. The precleaned lysate was incubated overnight with antibody-bound beads. The column was placed in a collection tube, and flowthrough was collected. The antigen antibody-bound beads were then subjected to washing with the washing and conditioning buffer. Bound antigen was eluted using elution buffer and further subjected to the Western blot analysis. All buffer used was provided in the kit.

### Indirect immunofluorescence assay.

*Plasmodium* cultures were grown (6% parasitemia) with synchronous stage-specific cultures at the ES, MS, and LS stages, washed with 1× PBS, and subsequently fixed using 4% paraformaldehyde for 15 min. Subsequently, the culture was washed with PBS and permeabilized using a 1:3 ratio of ice-cold acetone and methanol mixture for 15 min. Bovine serum albumin (BSA [3%]) was used as the blocking solution; subsequently, anti-mouse anti-cytochrome *c* (Abcam), anti-rabbit anti-TopoVIB (9), and anti-rabbit anti-Spo11 primary antibodies were used at 1:50, 1:25, and 1:25 dilutions, respectively, to probe the parasites for 1 h at 37°C. Slides were washed 3 times using 1× PBS, 1× PBS-Tween 20 (PBST), and 1× PBS for 15 min each. The secondary antibody cocktail containing Alexa Fluor 488-conjugated goat anti-mouse IgG (green) and Alexa Fluor 594-conjugated chicken anti-rabbit IgG (red) at a 1:250 dilution and DAPI (blue) (Invitrogen) at a 1:50 dilution was used and successively washed three times. At the end, parasites were mounted with antifade (Life Technologies). Nikon Eclipse NiE AR fluorescence microscope was used for analyzing and capturing the green and red fluorescence of PfCytc and PfTopoVIB/PfSpo11, respectively. The Pearson correlation coefficient (PCC) and the Manders’ overlap coefficient (MOC) were calculated for immunofluorescence antibody (IFA) images using the JaCoP plugin in ImageJ (v.1.52s) software.

### Fixed-ratio isobologram method to determine interaction between radicicol and atovaquone.

To understand the *in vitro* interaction between radicicol (Sigma) and atovaquone (Sigma), a fixed-ratio isobologram method was used. The IC_50_ of radicicol was determined by treating the synchronous schizont stage-specific 3D7 parasites with different concentrations of radicicol for 48 h at 37°C. In order to determine the IC_50_ of atovaquone, synchronous trophozoite-specific 3D7 parasites were treated with different concentrations of atovaquone for 48 h at 37°C. Both Giemsa and SYBER green-based staining (using a plate reader) were used to determine the percentage of parasite inhibition. The IC_50_ was calculated by plotting the percentage of inhibition against the concentration of the drug on a semilog graph using GraphPad Prism 6.

To check the interaction between radicicol and atovaquone, we used the protocol described in reference 23. Radicicol and atovaquone were combined in four fixed ratios (4:1, 3:2, 2:3, and 1:4). The effects of each of the drug combinations (along with their 2-fold serial dilutions) on the development of the parasite were assayed in triplicate. A 96-well plate was used, in which each well contained a total of 200 μL of reaction volume with 100 μL of culture (1% parasitemia and 5% hematocrit) and 100 μL of medium with or without drug. After setting the reaction, plates were incubated at 37°C for 48 h, followed by an assessment of parasite count by the SYBR green I-based method. The IC_50_ for each combination was calculated by plotting the semilog graph. The fractional inhibitory concentration (FIC) for each drug was determined by using the equation FIC = IC_50_ of the drug in mixture/IC_50_ of the drug alone. The interaction between radicicol and atovaquone was identified by computing ΣFIC, which was determined by using the formula ΣFIC = (IC_50_ of radicicol in mixture/IC_50_ of radicicol alone) + (IC_50_ of atovaquone in mixture/IC_50_ of atovaquone alone). The isobologram was prepared by using GraphPad Prism software. An ΣFIC value of <1 represents synergism, ΣFIC values of ≥1 and <2 represent additive interaction (no interaction), and an ΣFIC value of ≥2 represents antagonism. In a similar way, the interaction between radicicol and chloroquine (Sigma) was determined.

### Sequence- and structure-based comparison of PfTopoVIB and HsTopoVIBL.

The sequences of TopoVIB/TopoVIBL proteins were retrieved from the UniProt database (24). The UniProt ID numbers for *S. shibatae*, P. falciparum, Plasmodium berghei, Mus musculus, and Homo sapiens were O05207, Q8ID53, A0A509AMZ8, J3QMY9, and Q8N6T0, respectively. A multiple-sequence alignment was performed using ClustalV (25). The structures of PfTopoVIB and HsTopoVIBL were retrieved from the AlphaFold database (26). Structure alignment was performed using VMD (27).
