# Supplementary figures and images for: Plasmodium Topoisomerase VIB and Spo11 Constitute Functional Type IIB Topoisomerase in Malaria Parasite: Its Possible Role in Mitochondrial DNA Segregation
Source: Microbiol Spectr. 2023 May 22;11(3):e04980-22. doi: 10.1128/spectrum.04980-22 (PMC10269783; doi:10.1128/spectrum.04980-22)

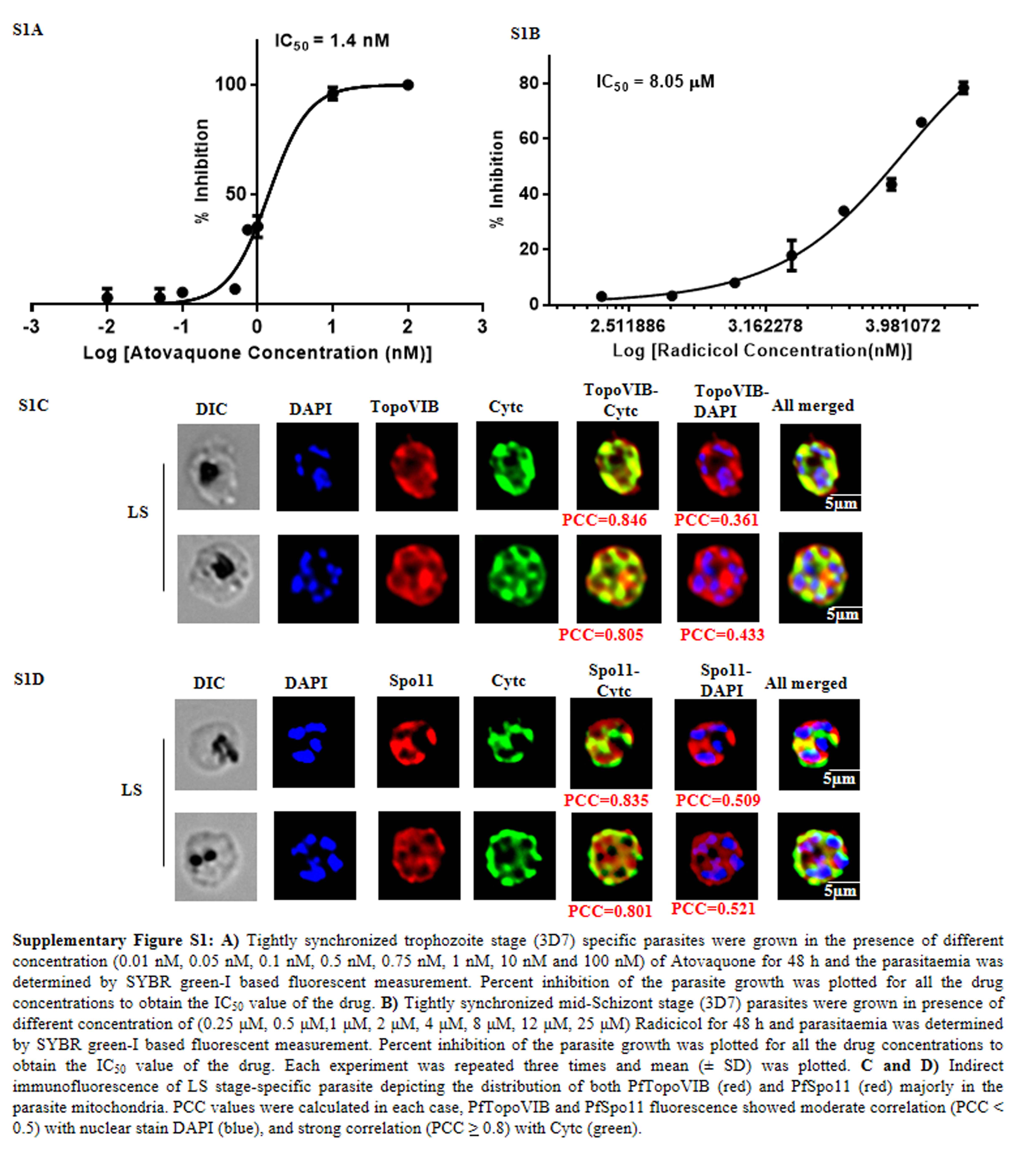

Supplement: Supplemental file 1 — Fig. S1. Download spectrum.04980-22-s0001.tif, TIF file, 10.8 MB [file spectrum.04980-22-s0001.tif]
